# Nutritional, biochemical and sensory properties of instant beverage powder made from two different varieties of pearl millet

**DOI:** 10.29219/fnr.v62.1524

**Published:** 2018-11-23

**Authors:** Anthony O. Obilana, Barthi Odhav, Victoria A. Jideani

**Affiliations:** 1 Food Technology Department, Cape Peninsula University of Technology, Bellville Campus, Cape Town, South Africa; 2 Biotechnology and Food Technology Department, Durban University of Technology, Durban, South Africa

**Keywords:** Instant beverage powder, pearl millet, combination processing, malting, extrusion, Instant beverage powder

## Abstract

**Introduction:**

The traditional method of producing instant foods involves producing a gelatinised paste from the preferred grain flour and proceeding to dry it using a drum drier. This produced a flaked product, which can be used as is or ground and sieved to obtain the desired particle size. With the advent of extrusion cooking technology and diverse production processes associated with the technology, food products including instant foods from cereals were developed.

**Objectives:**

The primary objective of this study was to produce a nutritious and acceptable pearl millet instant beverage powder (PMIBP) using combination processing.

**Methods:**

The effect of different processing methods (malting, extrusion, and a combination of both processes) on the nutritional, biochemical, and sensory characteristics of beverage powders and beverages made from two varieties of pearl millet (Pennisetum glaucum) were evaluated.

**Results:**

Combination processing led to a significant (p ≤ 0.05) decrease in total fat and total dietary fibre (TDF) (3.85 and 22.99 g/100 g, respectively) of AgriGreen (AgG) extruded malted pearl millet (EMPM) and extruded raw pearl millet–malted pearl millet mix (ERPMMPM). Combination processing also led to a decrease in the ash, total fat, TDF, Fe and Zn content (1.76, 3.48, 14.26 g/100 g, 7.78 and 4.74 mg/100 g, respectively) of Babala (Ba) EMPM and Ba ERPMMPM (1.88, 4.22, 21.71 g/100 g, 7.24 and 4.14 mg/100 g, respectively). Beverages of 10% total solids were prepared from the samples and offered to an untrained consumer panel. The beverages were rated on appearance, colour, aroma, flavour, texture and overall acceptability on a nine-point hedonic scale. In general, Ba raw pearl millet was rated 4 (like slightly), AgG malted pearl millet was rated 6 (dislike slightly), and all other pearl millet samples from both varieties were rated 5 (neither like nor dislike).

**Conclusion:**

Although combination processing led to an increase in carbohydrates, Ca, energy, Fe content, and 12 of the 15 amino acids measured as well as protein and starch digestibility and no change in the other nutrients measured, this did not significantly impact on the acceptability of the beverages.

Combination processing (hurdle technology) is the use of two or more processing methods in the manufacture and preservation of a food product. It was initially developed in order to ensure microbiological food safety. However, this concept is proving successful as an intelligent combination of hurdles secures microbial stability and safety as well as the sensory quality of foods, provides convenience and freshness of foods to the consumers, and might be cost-efficient for the producers because it demands less energy during production and storage (1).

Babala (Ba) is the most widely used variety of pearl millet in the world. It has been developed to adapt to the specific climates in which it grows. It is a nutrient-dense grain with a variety of food and beverage applications. Hybrids of Ba are developed for various other climatic and weather conditions and, ideally, developed hybrids are required to have similar characteristics to Ba under various processing conditions. The objective of this study was to evaluate the effect of malting, extrusion, and a combination of both methods on the nutritional and biochemical properties and sensory characteristics of flours and their beverages made from two varieties of pearl millet (Ba and AgriGreen [AgG] – a hybrid of Ba).

## Materials and methods

### Source of materials

Two different varieties of pearl millet (*Pennisetum glaucum*), Ba and AgG, a hybrid of Babala, were obtained from Agricol Pty. Ltd., Cape Town, South Africa. All chemical reagents were obtained from Sigma-Aldrich South Africa. All equipment used was located in the Department of Food Technology, Cape Peninsula University of Technology (CPUT), Bellville, South Africa, and The Council for Scientific and Industrial Research (CSIR), Pretoria, South Africa.

### Processing of pearl millet into beverage powder

**Fig. 1:**
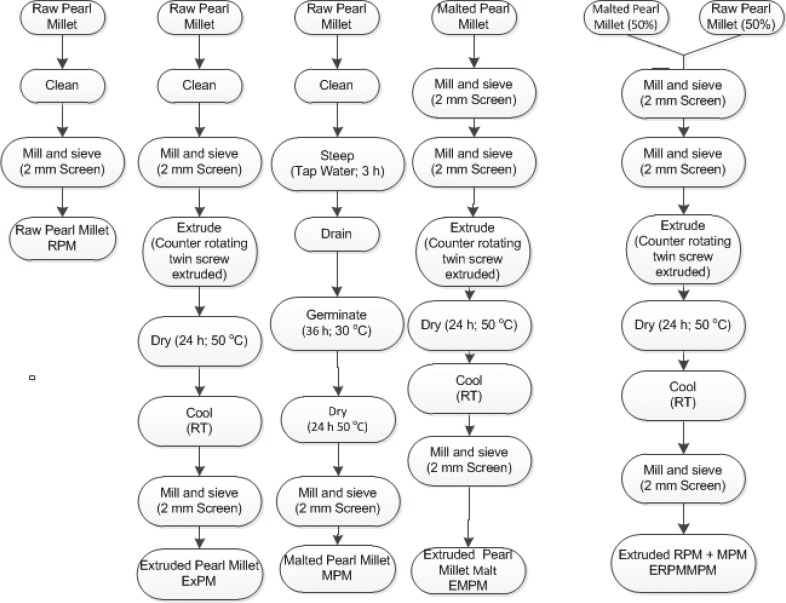
Processing steps of pearl millet into beverage. RPM = raw pearl millet, MPM = malted pearl millet, ERPMMPM = extruded mix of raw and malted pearl millet, RT = Room Temperature, EMPM = extruded pearl millet malt, ExPM = Extruded pearl millet.

The pearl millet (Ba and AgG) was cleaned by placing it in a tray and removing the chaff and damaged grains as well as stones or pebbles, together with all other extraneous matter, by a combination of winnowing and picking. The cleaned grains were further processed (Fig. 1).

### Determination of the proximate composition of pearl millet flours made from two different varieties

The moisture, protein, total ash, crude fibre contents, total fat, saturated, monounsaturated, and polyunsaturated fat content of the samples were determined according to the methods of the Association of Official Agricultural Chemists (AOAC) (2).

### Biochemical assay of beverage powder from two varieties of pearl millet

The amino acid content of the millet-based instant beverage powder was determined according to the methods of Benson and Patterson (3) and Klapper (4), with slight modifications. Calcium, iron, and zinc were analysed using the inductively coupled plasma (ICP) spectrometer (Perkin Elmer, model nr, Rodgau, Germany). Prior to analysis, samples were digested in a microwave digester (Milestone Microwave Laboratory Systems, Sorisole, Italy). The concentrations of minerals were calculated using the concentrations from the ICP analysis reports, using the following formula:

$$Mineral\;concentration\left(mg100g^{-1}\right)=\frac{Instrumentconcentration\left(ppm\right)\times Volume\left(ml\right)\times 100}{Massofsample\left(mg\right)}$$

Sample solutions were quantified against standard solutions of known concentrations that were analysed concurrently (5).

The determination of *in vitro* protein and starch digestibility was carried out according to the method of Saunders et al. (6). Digestibility was calculated using the following formula (7):

$$Proteindigestibility\left(\%\right)=\frac{Nitrogeninsupernatant\times 100}{Nitrogeninsample}$$

*In vitro* carbohydrate digestibility was determined using a method described by the authors of Ref. (8). Microsoft Excel (2010) was used to plot the standard curve and to calculate the concentration of starch digestion products in test solutions. The values were expressed as mg maltose/g starch.

The total amount of phenolic compounds in the pearl millet whole meal flour and product extract was determined using the method described by Silvia et al. (9) with modifications for use with a 96-well plate reader. The concentration of phenolic compounds in the extracts was calculated from a calibration curve of the standard and expressed as gallic acid equivalents.

The antioxidant activity (by free radical scavenging) of the pearl millet whole meal flour and products were determined using the Trolox (6-Hydroxy-2,5,7,8-tetramethylchroman-2-carboxylic acid) equivalent antioxidant capacity (TEAC) assay as described by the authors of Ref. (10) with modifications for a 96-well plate reader.

### Beverage preparation and sensory evaluation

The sensory evaluation of the beverages was exploratory in nature in order to determine if any one of the samples warranted further development. The flour (80 g) of each sample (raw pearl millet [RPM], extruded pearl millet [ExPM], malted pearl millet [MPM], extruded pearl millet malt [EMPM], and extruded mix of raw and malted pearl millet [ERPMMPM]) were individually weighed into separate 3 L stainless steel pots with 200 ml of tap water (25 ± 3°C) and mixed to form a paste. Boiling water (700 ml) was then added slowly whilst stirring to prevent the formation of lumps. Two 900 ml pots of each sample beverage were prepared to give a total of 1800 ml of beverage for each sample. The mixture was brought to a rolling boil. The prepared beverages were allowed to cool to between 50 and 60°C and then transferred into appropriately labelled 18/8 stainless steel double-walled vacuum thermal flasks.

The consumer panel assessments were conducted at the CPUT Department of Food Science and Technology sensory facilities using an untrained panel consisting of a mix of staff and students of the department (78).

For each sample, 78 randomly generated three-digit numbers were used to code samples presented to the consumer. Freshly prepared beverages of each sample (15–25 ml) were poured into 30 ml polystyrene cups to retain temperature (50–60°C) and consistency during the evaluation. Each consumer was presented with five cups of beverages on a polystyrene tray representing the five samples at between 40 and 45°C. The consumers were provided with water and an empty polystyrene cup to use as a spittoon and were instructed to rinse their mouths between samples. They were given written instructions together with a nine-point hedonic scale (1 = like extremely to 9 = dislike extremely), on which they were required to rate each sample’s flavour, texture, taste, colour, and overall acceptability.

### Data analyses

All data were collected in triplicate. The data were subjected to multivariate analysis of variance to establish mean differences (*p* ≤ 0.05) between treatments. Duncan multiple range tests were used to separate means where differences existed. All data analyses were carried out using IBM SPSS Statistics version 21 (2012).

## Results and discussion

### Effect of malting, extrusion, and their combination on the proximate content of beverage powders made from two varieties of pearl millet

Malting, extrusion, and the combination of both had varying effects on the nutritional values of RPM, ExPM, MPM, EMPM, and ERPMMPM produced from both varieties of pearl millet (Table 1). The effect of extrusion on nutritional values (Table 1) include the following: a significant (*p* ≤ 0.05) increase in the total fats (3.98 to 4.61 g/100 g); ash (1.75 to 2.03 g/100 g); carbohydrates (81.64 to 83.56 g/100 g); energy (1723.80 to 1789.44 KJ/100 g); and the minerals (Table 2) Ca (35.05 to 36.23 mg/100 g) and Fe (7.10 to 9.63 mg/100 g).

**Table 1:** Effect of processing on the proximate composition (g/100 g) and energy (KJ/100 g) of pearl millet (AgriGreen and Babala) (d.b.)^1^,^2^

	Nutrient	RPM	ExPM	MPM	EMPM	ERPMMPM
AgriGreen	Moisture	12.56 ± 0.25^a^	9.68 ± 0.21^b^	9.60 ± 0.03^b^	8.41 ± 0.15^c^	9.47 ± 0.07^b^
	Protein	12.46 ± 0.22^a^	12.30 ± 0.28^a^	12.73 ± 0.64^a^	12.51 ± 0.16^a^	12.47 ± 0.09^a^
	Ash	1.75 ± 0.11^a^	2.03 ± 0.14^b^	1.54 ± 0.32^a^	1.67 ± 0.09^a^	1.75 ± 0.02^a^
	Total fat	3.98 ± 0.41^a^	4.61 ± 0.30^b^	2.93 ± 0.29^c^	3.85 ± 0.19^a^	4.21 ± 0.08^a^
	TDF	26.59 ± 3.15^a^	17.11 ± 0.75^b^	19.33 ± 1.99^c^	22.99 ± 2.90^c^	18.12 ± 2.62^b^
	Carbohydrates	81.64 ± 0.34^a^	83.56 ± 0.90^b^	85.41 ± 0.64^c^	85.68 ± 0.35^c^	84.27 ± 0.06^b^
	Energy	1723.80 ± 5.48^a^	1789.44 ± 3.14^b^	1763.19 ± 11.53^c^	1804.34 ± 2.78^d^	1789.83 ± 3.48^b^
Babala	Moisture (g/100 g)	11.91 ± 0.06^a^	6.88 ± 0.04^b^	10.69 ± 0.11^c^	8.18 ± 0.06^d^	7.76 ± 0.19^e^
	Protein (g/100 g)	12.03 ± 0.18^a^	12.06 ± 0.08^a^	12.75 ± 0.04^b^	12.36 ± 0.26^c^	12.46 ± 0.11^c^
	Ash (100/g)	1.98 ± 0.04^a^	1.97 ± 0.03^a^	1.83 ± 0.07^b^	1.76 ± 0.07^b^	1.88 ± 0.12^b^
	Total fat (g/100 g)	4.79 ± 0.17^a^	4.25 ± 0.50^b^	2.84 ± 0.56^c^	3.48 ± 0.37^c^	4.22 ± 0.55^b^
	TDF (g/100 g)	26.69 ± 4.58^a^	16.51 ± 0.53^b^	25.17 ± 7.82^c^	14.26 ± 2.15^b^	21.71 ± 5.89^c^
	Carb (g/100 g)	81.64 ± 0.09^a^	86.85 ± 0.48^b^	84.17 ± 0.62^c^	86.35 ± 0.63^d^	85.73 ± 0.41^d^
	Energy (KJ/100 g)	1750.11 ± 1.99^a^	1838.08 ± 12.42^b^	1735.04 ± 10.60^a^	1799.15 ± 8.42^c^	1821.70 ± 13.71^d^

1 Values are mean ± standard deviation. Different superscripts in rows differ significantly (*p* ≤ 0.05).

2 RPM = raw pearl millet; ExPM = extruded pearl millet; MPM = malted pearl millet; EMPM = extruded pearl millet malt; ERPMMPM = extruded raw pearl millet–malted pearl millet mix; TDF = total dietary fibre.

**Table 2:** Effect of processing on the mineral (mg/100 g) composition of pearl millet (AgriGreen and Babala) (d.b.)^1^,^2^

	Nutrient	RPM	ExPM	MPM	EMPM	ERPMMPM
AgriGreen	Ca	35.05 ± 0.25^a^	36.23 ± 0.04^b^	38.78 ± 0.45^c^	40.32 ± 0.25^d^	36.90 ± 0.14^e^
	Fe	7.10 ± 0.29^a^	9.63 ± 0.29^b^	7.01 ± 0.16^a^	8.56 ± 0.28^c^	10.57 ± 0.31^d^
	Zn	3.43 ± 0.13^a^	3.30 ± 0.08^a^	4.18 ± 0.09^b^	3.19 ± 0.11^a^	3.16 ± 0.50^a^
Babala	Ca	30.74 ± 0.25^a^	27.43 ± 0.20^b^	34.15 ± 0.13^c^	32.56 ± 0.24^d^	33.66 ± 0.28^d^
	Fe	9.60 ± 0.43^a^	9.51 ± 0.09^a^	7.08 ± 0.45^b^	7.78 ± 0.13^c^	7.24 ± 0.33^b^
	Zn	5.36 ± 0.54^a^	5.51 ± 0.37^a^	3.97 ± 0.45^b^	4.74 ± 0.05c^b^	4.14 ± 0.08^b^

1 Values are mean ± standard deviation. Different superscripts in rows differ significantly (*p* ≤ 0.05).

2 RPM = raw pearl millet; ExPM = extruded pearl millet; MPM = malted pearl millet; EMPM = extruded pearl millet malt; ERPMMPM = extruded raw pearl millet–malted pearl millet mix.

These changes depend on temperature, moisture, pH, shear rate, residence time, their interactions, the nature of the proteins themselves, and the presence of materials such as carbohydrates and lipids (11). The time–temperature conditions to which foods are exposed during extrusion are comparable to other high-temperature, short-time processes, which is considered preferable in terms of nutrient retention and safety of foods since antinutritional factors and contaminating microorganisms are more effectively destroyed (12).

According to Bjork and Asp (12), extrusion processing affects the nutritional value of lipids through different mechanisms such as oxidation, cis–trans isomerisation, or hydrogenation. A decrease in the fat content of extruded products has been reported by several authors. Fabriani et al. (13) interpreted the decrease in the extractable-fat content of extruded products as the result of the formation of complexes with other compounds present in the food matrix and/or shear damage caused by the action of the screws and subsequent pressures generated. These could explain the decrease in fat content observed in Babala. However, the increase in the extractable fat content of AgG (Table 1) could have been a result of the exact opposite happening, that is, no complexes being formed with other compounds in the food matrix during the processes and/or little or no shear damaged caused by the actions of the screws and subsequent pressures generated.

Malting led to a significant (*p* ≤ 0.05) increase in the carbohydrates, energy (Table 1), Ca, and Zn (Table 2) (81.64 to 85.41 g/100 g, 1723.8 to 1763.2 KJ/100 g, 35.05 to 38.78 mg/100 g and 3.43 to 4.18 mg/100 g, respectively). A significant (*p* ≤ 0.05) decrease in the TDF and total fat (26.59 to 19.33 and 3.98 to 2.93 g/100 g respectively) was observed, but no effect on the protein, ash, and Fe content of AgG MPM (Table 1). These are in contrast to observations of slightly increased protein content (11, 7, and 2%, respectively) for red sorghum, millet, and maize made by Traoré et al. (14). Shayo et al. (15) also observed an increase in protein content of 5% after 48 h of germination at 30°C in two varieties of millet from Tanzania. Whilst the increase in protein content in these experiments was attributed to a passive variation resulting from a decrease in the carbohydrate compounds used for respiration (16), the lack of change in protein content in this particular experiment could be attributed to the shorter germination time (36 h as opposed to 48 h). According to Chavan and Kadam (17), a considerable portion of endosperm carbohydrates decrease during germination, causing apparent increase in the protein and fibre contents of cereals; this could be the reason for no marked changes in the endosperm protein content of sorghum and pearl millet, although the rootlets separated from them contained substantial levels of protein.

The decreases in fat content are in agreement with observations made by other authors (14, 16–18). This decrease could be explained by the fact that lipids are used to produce the necessary energy for the biochemical and physiological modifications that occur in the seed during germination (18). Combination processing (malting and extrusion) led to a significant (*p* ≤ 0.05) increase in carbohydrates, energy (Table 1), Ca, and Fe (Table 2) (81.64 to 85.68 g/100 g, 1723.8 to 1804.3 KJ/100 g, 35.05 to 40.32 and 7.10 to 8.56 mg/100 g, respectively); a significant (*p* ≤ 0.05) decrease in TDF (26.59 to 22.99 g/100 g); and no effect on the protein and ash content of AgG EMPM.

Combination processing of the ERPMMPM led to a significant (*p* ≤ 0.05) increase in carbohydrates, energy (Table 1), Ca, and Fe (Table 2) (81.64 to 84.27 g/100 g, 1723.8 to 1789.8 KJ/100 g, 35.05 to 36.90 and 7.10 to 10.57 mg/100 g, respectively); a significant (*p* ≤ 0.05) decrease in TDF (26.59 to 18.12 g/100 g); and no effect on ash, total fat, and Zn content of AgG ERPMMPM.

The extrusion process led to a significant (*p* ≤ 0.05) increase in carbohydrates and energy (81.64 to 86.85 g/100 g and 1750.11 to 1838.08 KJ/100 g, respectively); a significant (*p* ≤ 0.05) decrease in total fat, TDF (Table 1), and Ca (Table 2) (4.79 to 4.25, 26.69 to 16.51 g/100 g and 30.74 to 27.43 mg/100 g, respectively); but had no effect on the protein, ash, Fe, and Zn content of Ba ExPM (Table 1).

The observations on the effect of malting on proximate composition, mineral (Ca, Fe, and Zn) and fibre content of both AgG and Ba were in agreement with observations made by several authors (19–21) but differed from observations made by Opoku et al. (16) and Suma and Urooj (22). According to Malleshi and Klopfenstein (21), during germination several biochemical, textural, and physiological transformations occur in the seeds. The growing root and shoot mainly derive nutrients from the embryo, scutellum, and the endosperm and this result in loss of protein, carbohydrates, and minerals from the seed. Consequently, the proportion of some of these nutrients in the malt will be altered.

Leaching of water-soluble compounds and metabolism of carbohydrates during germination also contribute to dry matter loss of seeds. This could explain the varying changes in the nutritional properties of the pearl millet after malting.

Malleshi and Klopfenstein (21) observed that raw sorghum and pearl millet contained 11.8 and 16.1% protein, respectively, which did not change appreciably on malting and is in agreement with the observations made for the protein content of AgG but differed for that of Ba. They also observed a slight increase in the dietary fibre content of their samples after malting.

This was in contradiction to observations made in this experiment. Also, dietary fibre levels reported in their works were markedly lower than those reported in this work. Combination processing (malting and extrusion) led to a significant (*p* ≤ 0.05) increase in protein content, carbohydrates, energy (Table 1), and Ca (Table 2) (12.03 to 12.36, 81.64 to 86.35 g/100 g, 1750.11 to 1799.2 KJ/100 g and 30.74 to 32.56 mg/100 g, respectively) and a significant (*p* ≤ 0.05) decrease in the ash, total fat, TDF (Table 1), Fe, and Zn (Table 2) (1.98 to 1.76, 4.79 to 3.48, 26.69 to 14.26 g/100 g, 9.60 to 7.78 and 5.36 to 7.74 mg/100 g, respectively) content of the EMPM. Combination processing of the ERPMMPM led to a significant (*p* ≤ 0.05) increase in the protein, carbohydrates, energy (Table 1), and Ca (Table 2) (12.03 to 12.46, 81.64 to 85.73 g/100 g, 1750.11 to 1821.70 KJ/100 g and 30.74 to 33.66 mg/100 g) and a significant (*p* ≤ 0.05) decrease in the ash, total fat, TDF (Table 1), Fe, and Zn (Table 2) (1.98 to 1.88, 4.79 to 4.22, 26.69 to 21.71 g/100 g, 9.60 to 7.24 and 5.36 to 4.14 mg/100 g, respectively) content of Ba ERPMMPM. These variations in values observed could be attributed to several factors such as differences in the pearl millet varieties experimented with as well as extrinsic factors including growth region, climate, and soil type, to name a few. The decrease in moisture content of both AgG and Ba was the result of the kilning of germinated grains. The information observed is a summary and a reinforcement of earlier discussions on the effect of the processing methods on the two varieties of pearl during the production of the Pearl Millet Instant Beverage Powder (PMIBP).

Extrusion, malting, and a combination of both processes significantly (*p* ≤ 0.05) reduced the fat constituents (saturated, monounsaturated, and polyunsaturated) of Babala (Table 3). However, extrusion significantly (*p* ≤ 0.05) increased the content of polyunsaturated fat of AgG, but the combination process did not affect the fat constituents of AgG significantly (*p* ≤ 0.05) (Table 3). The reduction in fat content could be as a result of conversion or utilisation for energy during germination or through different mechanisms such as oxidation, cis–trans isomerisation, or hydrogenation during extrusion processing (12).

**Table 3:** Effect of processing on the fat constituents (g/100 g) of pearl millet (AgriGreen and Babala) (d.b.)^1^,^2^

	Nutrient	RPM	ExPM	MPM	EMPM	ERPMMPM
AgriGreen	Total fat (g/100 g)	3.98 ± 0.41^a^	4.61 ± 0.30^b^	2.93 ± 0.29^c^	3.85 ± 0.19^a^	4.21 ± 0.08^a^
	Saturated fat (g/100 g)	1.11 ± 0.10^a^	1.27 ± 0.06^b^	0.83 ± 0.07^c^	1.07 ± 0.05^a^	1.16 ± 0.03^a^
	Monounsaturated fat (g/100 g)	1.14 ± 0.13^a^	1.33 ± 0.10^b^	0.82 ± 0.09^c^	1.09 ± 0.07^a^	1.23 ± 0.02^a^
	Polyunsaturated fat (g/100 g)	1.72 ± 0.18^a^	2.01 ± 0.14^b^	1.28 ± 0.13^c^	1.69 ± 0.08^a^	1.82 ± 0.05^a^
Babala	Total fat (g/100 g)	4.79 ± 0.17^a^	4.25 ± 0.50^b^	2.84 ± 0.56^c^	3.48 ± 0.37^c^	4.22 ± 0.55^b^
	Saturated fat (g/100 g)	1.24 ± 0.06^a^	1.16 ± 0.14^b^	0.78 ± 0.15^c^	0.96 ± 0.11^c^	1.15 ± 0.16^b^
	Monounsaturated fat (g/100 g)	1.28 ± 0.04^a^	1.18 ± 0.20^b^	0.76 ± 0.20^c^	0.95 ± 0.15^c^	1.16 ± 0.22^b^
	Polyunsaturated fat (g/100 g)	2.27 ± 0.11^a^	1.92 ± 0.16^b^	1.29 ± 0.21^c^	1.57 ± 0.10^c^	1.92 ± 0.17^b^

1 Values are mean ± standard deviation. Different superscripts in columns differ significantly (*p* ≤ 0.05).

2 RPM = raw pearl millet; ExPM = extruded pearl millet; MPM = malted pearl millet; EMPM = extruded pearl millet malt; ERPMMPM = extruded raw pearl millet–malted pearl millet mix.

### Effect of malting, extrusion, and their combination on the amino acid content of beverage powders made from two varieties of pearl millet

The effects of malting, extrusion, and a combination of both methods on the amino acid content of the PMIBP made from the two different varieties of pearl millet, AgG and Ba, are shown in Table 4. For the AgG variety, extrusion led to a significant (*p* ≤ 0.05) increase in the concentration of the amino acids in the AgG ExPM.

**Table 4:** Effect of processing on the amino acid content (mg/100 g) of pearl millet (Babala and AgriGreen)^1^,^2^

	AA	RPM	ExPM	MPM	EMPM	ERPMMPM
AgriGreen	Asp	0.43 ± 0.03^a^	0.72 ± 0.03^b^	0.68 ± 0.04^b^	0.64 ± 0.03^b^	0.57 ± 0.06^c^
	Thr	0.22 ± 0.00^a^	0.33 ± 0.02^b^	0.29 ± 0.00^c^	0.26 ± 0.00^d^	0.25 ± 0.03^d^
	Ser	0.27 ± 0.00^a^	0.43 ± 0.03^b^	0.36 ± 0.02^c^	0.35 ± 0.04^c^	0.33 ± 0.02^c^
	Glu	1.12 ± 0.05^a^	1.61 ± 0.16^b^	1.23 ± 0.04^a^	1.30 ± 0.14^a^	1.29 ± 0.14^a^
	Gly	0.19 ± 0.00^a^	0.29 ± 0.02^b^	0.25 ± 0.01^c^	0.23 ± 0.01^c^	0.23 ± 0.02^c^
	Ala	0.40 ± 0.01^a^	0.59 ± 0.05^b^	0.51 ± 0.01^c^	0.51 ± 0.04^c^	0.48 ± 0.03^c^
	Val	0.28 ± 0.03^a^	0.46 ± 0.03^b^	0.39 ± 0.03^c^	0.39 ± 0.01^c^	0.38 ± 0.01^c^
	Met	0.06 ± 0.00^a^	0.11 ± 0.01^b^	0.05 ± 0.03^a^	0.10 ± 0.04^c^	0.09 ± 0.01^c^
	Ile	0.23 ± 0.00^a^	0.34 ± 0.02^b^	0.27 ± 0.00^c^	0.29 ± 0.03^c^	0.27 ± 0.02^c^
	Leu	0.53 ± 0.01^a^	0.79 ± 0.08^b^	0.59 ± 0.01^a^	0.63 ± 0.06^a^	0.63 ± 0.06^a^
	Tyr	0.21 ± 0.01^a^	0.32 ± 0.04^b^	0.27 ± 0.01^c^	0.28 ± 0.01^c^	0.25 ± 0.02^c^
	Phe	0.28 ± 0.01^a^	0.42 ± 0.04^b^	0.35 ± 0.02^c^	0.35 ± 0.04^c^	0.32 ± 0.02^c^
	His	0.17 ± 0.00^a^	0.28 ± 0.04^b^	0.26 ± 0.01^b^	0.26 ± 0.04^b^	0.23 ± 0.02^b^
	Lys	0.19 ± 0.00^a^	0.32 ± 0.02^b^	0.32 ± 0.01^b^	0.31 ± 0.09^b^	0.24 ± 0.03^a^
	Arg	0.23 ± 0.01^a^	0.48 ± 0.06^b^	0.44 ± 0.13^b^	0.37 ± 0.08^a^	0.38 ± 0.02^a^
Babala	Asp	0.46 ± 0.05^a^	0.61 ± 0.04^b^	0.79 ± 0.00^c^	0.68 ± 0.03^c^	0.69 ± 0.02^c^
	Thr	0.23 ± 0.04^a^	0.27 ± 0.01^b^	0.27 ± 0.00^b^	0.29 ± 0.00^b^	0.28 ± 0.04^b^
	Ser	0.33 ± 0.04^a^	0.36 ± 0.01^a^	0.35 ± 0.00^a^	0.38 ± 0.01^a^	0.35 ± 0.00^a^
	Glu	1.23 ± 0.21^a^	1.45 ± 0.09^b^	1.11 ± 0.00^a^	1.42 ± 0.08^b^	1.43 ± 0.16^b^
	Gly	0.20 ± 0.03^a^	0.25 ± 0.02^b^	0.28 ± 0.00^b^	0.24 ± 0.00^b^	0.24 ± 0.00^b^
	Ala	0.47 ± 0.07^a^	0.54 ± 0.03^b^	0.44 ± 0.00^a^	0.54 ± 0.03^b^	0.53 ± 0.03^c^
	Val	0.36 ± 0.07^a^	0.40 ± 0.03^a^	0.38 ± 0.00^a^	0.42 ± 0.03^a^	0.40 ± 0.02^a^
	Met	0.09 ± 0.02^a^	0.08 ± 0.02^b^	0.05 ± 0.00^a^	0.12 ± 0.01^b^	0.12 ± 0.05^c^
	Ile	0.27 ± 0.04^a^	0.29 ± 0.01^b^	0.26 ± 0.00^a^	0.33 ± 0.02^b^	0.33 ± 0.02^b^
	Leu	0.62 ± 0.11^a^	0.71 ± 0.03^b^	0.55 ± 0.00^a^	0.72 ± 0.03^b^	0.69 ± 0.04^b^
	Tyr	0.24 ± 0.02^a^	0.28 ± 0.02^b^	0.25 ± 0.00^a^	0.32 ± 0.01^b^	0.28 ± 0.01^b^
	Phe	0.33 ± 0.04^a^	0.39 ± 0.02^a^	0.33 ± 0.00^a^	0.39 ± 0.01^a^	0.38 ± 0.07^a^
	His	0.29 ± 0.06^a^	0.24 ± 0.00^a^	0.29 ± 0.00^a^	0.26 ± 0.01^a^	0.36 ± 0.08^b^
	Lys	0.22 ± 0.02^a^	0.29 ± 0.09^a^	0.32 ± 0.00^a^	0.30 ± 0.09^a^	0.28 ± 0.03^a^
	Arg	0.35 ± 0.06^a^	0.35 ± 0.04^a^	0.36 ± 0.00^a^	0.83 ± 0.37^b^	0.39 ± 0.17^a^

1 Values are mean ± standard deviation. Different superscripts in columns differ significantly (*p* ≤ 0.05).

2 RPM = raw pearl millet; ExPM = extruded pearl millet; MPM = malted pearl millet; EMPM = extruded pearl millet malt; ERPMMPM = extruded raw pearl millet–malted pearl millet mix; AA = Amino Acid.

Malting led to a significant (*p* ≤ 0.05) increase in all amino acids, except for glutamic acid, leucine, and methionine, which remained unchanged in the AgG MPM. The combination of malting and extrusion significantly (*p* ≤ 0.05) increased all amino acids except for glutamic acid, leucine, and arginine in the AgG EMPM. Similar results were obtained for the AgG ERPMMPM, with glutamic acid, leucine, lysine, and arginine remaining unchanged by the process. None of the treatments led to a decrease in the amino acid content of AgG. Similar results were obtained for Ba with a different set of amino acids remaining unchanged in the RMF and extruded samples.

Germination of cereals is known to increase their lysine and tryptophan contents. The subject has been reviewed exhaustively by the authors of Refs. (17) and (23). However, Malleshi and Klopfenstein (21) only observed similar trends in finger millet as its lysine content increased on malting, but no appreciable changes in the lysine content were observed during sorghum and pearl millet germination. Elmalik et al. (24) reported an increase in most of the amino acid contents of sorghum cultivars of varying endosperm texture on germination and the increase being higher in corneous cultivars than the floury cultivars.

According to Malleshi and Klopfenstein (21), malting of sorghum and millets marginally enhances some of their essential amino acids but substantially improves their riboflavin, niacin, and ascorbic acid contents. Malting in combination with extrusion led to either a significant (*p* ≤ 0.05) increase or no change in the amino acid content.

This is promising as it means that there would not be a need for replacement fortification of the finished product with the lost amino acids. Of the nine essential amino acids required by humans, seven were identified in the ERPMMPM of both varieties of pearl millet; valine and tryptophan were not identified in the various samples.

The amino acid content of pearl millet was lower than the Nutrient Reference Values (NRV). This was observed by other researchers and informed the decision to composite millet especially with legumes in order to increase their content in the resultant complementary food, and hence its protein quality, *viz*. protein digestibility corrected amino acid score. The NRVs are based on Recommended Dietary Allowances (RDAs), which will meet the needs of nearly all (97 to 98%) healthy individuals to prevent nutrient deficiencies. RDA values are not necessarily enough to maintain optimum nutritional status and prevent chronic disease. These values are therefore considered the minimum amounts necessary to achieve and maintain optimum nutritional status, which will assist in the reduction of disease, specifically degenerative diseases of lifestyle (25). This could be happening for several reasons, including the degradation of antinutritional properties, the presence of which could prevent quantification of amino acids in the RPM. According to El-Hady et al. (26), soaking reduced phytic content (known antinutrients) in all tested legumes in their experiment. Their data were in agreement with the findings of Alonso et al. (27) and these reductions may be ascribed to the activation of the endogenous phytase during the long soaking treatment and possible enzyme action continued during the germination and drying steps of malting. They also observed a further decrease in the phytic content on extruding their samples at high (180°C) temperatures.

### Effect of malting, extrusion, and their combination on the in vitro protein and starch digestibility of beverage powders made from two varieties of pearl millet

Table 5 summarises the *in vitro* protein and starch digestibility for RPM, ExPM, MPM, EMPM, and ERPMMPM from both AgG and Ba. Malting significantly (*p* ≤ 0.05) decreased the *in vitro* protein (69.4%) and starch (33.24 mg maltose/100 g starch) digestibility of AgG, whilst extrusion had no effect on protein (73.18%) digestibility but significantly (*p* ≤ 0.05) increased the starch (66.90 mg maltose/100 g starch) digestibility of AgG.

**Table 5:** Digestibility of beverage powder produced from two varieties of pearl millet by malting, extrusion, and a combination of both processes^1^,^2^

	AgriGreen		Babala	
	Protein digestibility (%)	Starch digestibility (mg maltose/100 g starch)	Protein digestibility (%)	Starch digestibility (mg maltose/100 g starch)
RPM	87.20 ± 6.96^a^	40.01 ± 2.42^a^	96.61 ± 1.18^a^	35.44 ± 11.24^a^
ExPM	73.18 ± 10.90^a^	66.90 ± 18.37^b^	67.89 ± 5.10^b^	74.47 ± 9.55^b^
MPM	69.94 ± 5.16^b^	33.24 ± 6.13a	79.70 ± 4.66^c^	41.93 ± 2.54^a^
EMPM	70.04 ± 7.68^b^	65.70 ± 11.45^b^	69.45 ± 1.38^b^	41.56 ± 18.03^a^
ERPMMPM	61.81 ± 9.02^b^	65.85 ± 12.76^b^	78.29 ± 0.96^c^	37.95 ± 6.85^a^

1 Values are mean ± standard deviation. Different superscripts in columns differ significantly (*p* ≤ 0.05).

2 RPM = raw pearl millet; ExPM = extruded pearl millet; MPM = malted pearl millet; EMPM = extruded pearl millet malt; ERPMMPM = extruded raw pearl millet–malted pearl millet mix.

Combination processing led to a significant (*p* ≤ 0.05) decrease in protein digestibility of products from both AgG and Ba, a significant (*p* ≤ 0.05) increase in the starch digestibility of EMPM from AgG, and no change in the starch digestibility of EMPM from Ba.

Heat treatment of foods may enhance *in vitro* protein digestibility of food products by altering and breakdown of high molecular weight protein or by destroying the heat labile protease inhibitors. The increase in protein digestibility on malting could also be attributed to the degradation of storage protein (28), which may be more easily available to pepsin attack.

The proteins present in the feed material may undergo structural unfolding and/or aggregation when subjected to heat or shear during extrusion. Intact protein structures represent a significant barrier to digestive enzymes; and the combination of heat and shear is a very efficient way of disrupting such structures. In general, denaturation of protein to random configurations improves nutritional quality by making the molecules more accessible to proteases and thus more digestible. This is especially important in legume-based foods that contain active enzyme inhibitors in the raw state (11).

Disulphide bonds are involved in stabilising the native tertiary configurations of most proteins. Their disruption and shearing can contribute to the breaking of these bonds, aiding in protein unfolding and thus digestibility (11).

Partial hydrolysis of proteins during extrusion increases their digestibility by producing more open configurations and increasing the number of exopeptidase-susceptible sites. Conversely, production of an extensively isopeptide cross-linked network could interfere with protease action, reducing the digestibility (29).

The inherent property of sprouting seeds to increase the hydrolytic activity of enzymes may cause the mobilisation of protein, leading to the formation of polypeptides, dipeptides, and free amino acids. Further, during malting, the polyphenols and phytic acids are catabolised, and in addition, their leftover amount was removed as malting loss. This may be responsible for increasing the protein digestibility during malting (30).

Considering that most investigators observed an increase in protein digestibility with processing, it is unclear what may have caused the opposite in these experiments, but there are some possible explanations that would need further investigation; the difference in types and varieties of raw materials (grains used) could be a factor in the difference in observations, as well as the processing (exact parameters) conditions for the experiments. It is possible that the combination of malting and extrusion, led to the formation of complexes between protein and other compounds thus lowering its digestibility. The decrease in *in vitro* protein digestibility for both AgG and Ba could be a direct result of an increase in total phenolic content after malting (Table 6).

**Table 6:** TPC and antioxidant activity of beverage powder from two varieties of pearl millet processed by malting, extrusion, and a combination of both processing methods^1^,^2^

	AgriGreen		Babala	
	Total phenolics (μg/g)	TEAC (μmole TE/g)	Total phenolics (μg/g)	TEAC (μmole TE/g)
RPM	2.67 ± 0.05^a^	1.80 ± 0.18^a^	3.04 ± 0.20^a^	1.73 ± 0.07^a^
ExPM	1.78 ± 0.06^b^	1.73 ± 0.18^a^	0.93 ± 0.05^b^	1.74 ± 0.18^a^
MPM	3.68 ± 0.04^c^	6.41 ± 0.30^b^	4.55 ± 0.12^c^	7.70 ± 0.10^b^
EMPM	1.34 ± 0.01^d^	2.14 ± 0.16^c^	1.30 ± 0.06^d^	1.78 ± 0.25^a^
ERPMMPM	1.59 ± 0.08^e^	2.59 ± 0.09^c^	1.62 ± 0.01^e^	3.34 ± 0.04^c^

OTE	2.34 ± 0.97	3.25 ± 2.06	2.28 ± 1.41	2.94 ± 2.15

1 Values are mean ± standard deviation. Different superscripts in columns differ significantly (*p* ≤ 0.05).

2 RPM = raw pearl millet; ExPM = extruded pearl millet; MPM = malted pearl millet; EMPM = extruded pearl millet malt; ERPMMPM = extruded raw pearl millet–malted pearl millet mix; OTE = overall treatment effect; TEAC = Trolox (6-Hydroxy-2,5,7,8-tetramethylchroman-2-carboxylic acid) equivalent antioxidant capacity; TE = Trolox Equivalent; TPC = Total phenolic content.

The *in vitro* starch digestibility for both AgG and Ba (Table 5) was improved significantly (*p* ≤ 0.05) by extrusion cooking, whilst malting only increased starch digestibility for Ba, with no effect on AgG; this is in agreement with the authors of Ref. 31, who also observed an increase in *in vitro* starch digestibility of pearl millet. This increase they attributed to malting loss, which may represent the removal of antinutrients present in sprouts. According to Holm et al. (32), other factors that have been shown to affect the starch digestion of food included degree of gelatinisation, granule particle size, amylose–mylopectin ratio, starch–protein interaction, amylase–lipid complexes, percentages of resistant or retrograded starch, and presence of other non-starch carbohydrates. In seeds, factors such as amylase inhibitors, phytic acid, and polyphenols have been reported to inhibit α-amylase (33, 34), hence decreasing *in vitro* starch digestibility. The levels of these compounds in pearl millet decreases during malting as a result of leaching and enzymatic breakdown; this in turn results in increased starch digestibility of malted pearl millet (31). During malting, amylase and phosphorylase might become active and catalyse amylolysis. The resulting increased concentration of oligosaccharides may contribute towards better starch digestibility of pearl millet malt (30).

### Effect of malting, extrusion, and their combination on the total phenolic content and antioxidant activity of beverage powders made from two varieties of pearl millet

The effects of extrusion and malting on the total phenolic content and antioxidant activity of AgrG and Ba are summarised in Table 6. Extrusion significantly (*p* ≤ 0.05) reduced total phenolic content of both AgG (1.78 μg/g) and Ba (0.93 μg/g), whilst malting significantly (*p* ≤ 0.05) increased total phenolic content of both AgG (3.68 μg/g) and Ba (4.55 μg/g). Extrusion had no effect on the antioxidant activity (TEAC) of AgG (1.73 μmole TE/g) and Ba (1.74 μmole TE/g), whilst malting significantly increased antioxidant activity (TEAC) of both AgG (6.41 μmole TE/g) and Babala (7.70 μmole TE/g).

Contrary to observations of the effect of malting on total phenolics (increase), Archana and Kawatra (31) reported polyphenol content in untreated (raw) pearl millet grains of 764.45 mg/100 g and observed a significant (*p* < 0.05) destruction of polyphenols by malting; the level of destruction was dependent on germination time.

It is speculated that leaching of polyphenols during steeping may account for some of this loss. Loss of polyphenols during malting may be attributed to the presence of polyphenol oxidase (25) and to the hydrolysis of tannin-protein and tannin-enzyme complexes, which results in the removal of tannins or polyphenols (37). Contrary to the present study, germination has been reported to reduce the polyphenol content in pearl millet (30, 38). The increase in total phenolics could be attributed to a possible increase in lignin (16).

### Sensory acceptability of the pearl millet–based instant beverage prepared from beverage powders made from two varieties of pearl millet

**Fig. 2:**
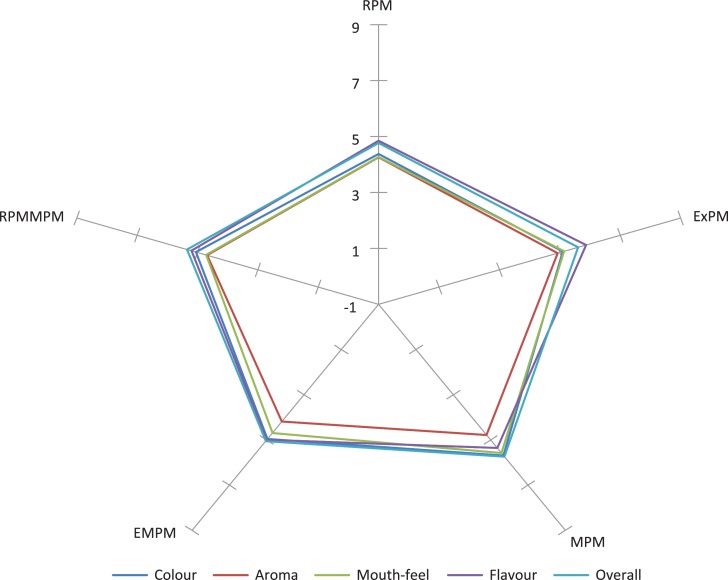
Spider sensory plot for Babala. RPM = raw pearl millet; ExPM = extruded pearl millet; MPM = malted pearl millet; EMPM = extruded pearl millet malt; ERPMMPM = extruded raw pearl millet–malted pearl millet mix.

**Fig. 3:**
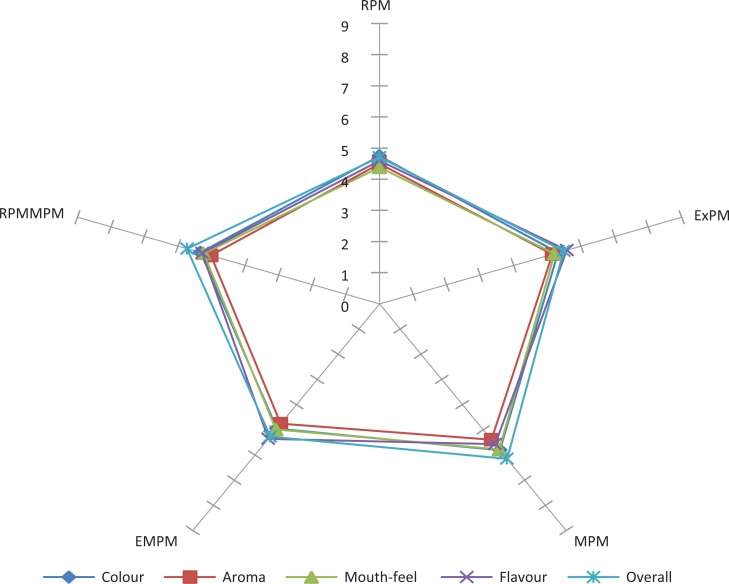
Spider sensory plot for AgriGreen. RPM = raw pearl millet; ExPM = extruded pearl millet; MPM = malted pearl millet; EMPM = extruded pearl millet malt, ERPMMPM = extruded raw pearl millet–malted pearl millet mix.

Figs. 2 and 3 summarise the sensory acceptability of RPM, ExPM, MPM, EMPM, and ERPMMPM beverages made from two varieties of pearl millet (AgG and Ba, respectively). The average overall acceptance rating for RPM, ExPM, MPM, EMPM, and ERPMMPM from Ba and AgG ranged from 4.71 ± 0.22 (like slightly) (AgG-RPM) to 6.15 ± 0.23 (dislike slightly) (AgG-RPM). In general, the different sensory attributes rated by the panellists ranged from ‘like slightly’ (=4) to ‘dislike slightly’ (=6).

The majority of the panellists neither liked nor disliked the different beverages. Significant differences (*p* ≤ 0.05) existed in all the panellists’ acceptability scores for the sensory attributes for the different products rated. The different backgrounds and possible prior exposure to similar products would affect the ratings of the different products (RPM, ExPM, MPM, EMPM, and ERPMMPM from pearl millet) by the panellists.

An improvement in the attributes of the beverages is required in order to improve and increase its overall acceptability. This can be achieved with significantly increased protein content and quality over the unsupplemented pearl millet by the addition of any of the following: soybean, morama bean, or Bambara groundnut. These would also act as functional ingredients supplying taste, texture, colour, and other properties to variety of foods (35). The percentage inclusion of the suggested legumes will have to be determined so as not to adversely affect the flavour and colour of the final product.

The colour difference calculated from the data collected (36) gives an indication of both the perception of a colour difference between ExPM, MPM, EMPM, ERPMMPM, and RPM and the effect of processing methods used for the preparation of the beverage powders.

The perception (visual) of a colour difference between samples could also be an influencing factor in rating of the other attributes of the beverages and hence the overall acceptability of the beverage.

### Conclusions

Beverages produced from both varieties of millet, though not unacceptable, were not acceptable to the panellists. Improving the colour or rather decreasing the colour difference (Δ*E*) as well as improving the flavour of the beverages could inevitably lead to better or increased overall acceptance of the beverages. These could be achieved by increasing the kilning temperature during malting, to affect the development of a more intense flavour profile as well as a roasted or toasted colour in the grains. Addition of suitable adjuncts could further boost the nutritional value of the products and, more importantly, increase the overall acceptability of beverages from pearl millet (AgG and Ba).
